# Nasotracheal intubation awake in a patient with multiple fractures of the maxilla and cervical spine: A case report

**DOI:** 10.1097/MD.0000000000034028

**Published:** 2023-06-09

**Authors:** Luan Oliveira Ferreira, Victoria Winkler Vasconcelos, Imaikon Gomes de Lima, Juliana Barbosa de Souza, Karina Dias Resende

**Affiliations:** a Programa de Residência Médica em Anestesiologia, Hospital Universitário João de Barros Barreto, Belém, Brasil; b Departamento de Anestesiologia, Hospital Metropolitano de Urgência e Emergência, Belém, Brasil.

**Keywords:** awake, cervical spine, general anesthesia, nasotracheal intubation

## Abstract

Nasotracheal intubation is an advanced airway modality used in specific contexts, such as limited mouth opening, macroglossia, cervical spine instability. In addition, it can be performed with the patient awake, especially when predictors a difficult airway are unknown.

**Patient concerns:**

A 41-year-old male with a lesion in the C1 cervical vertebra, associated with a fracture of the right maxilla, was intubated through the nasopharyngeal route while awake. The forms of induction were discussed.

**Diagnoses:**

Based on the mechanism of trauma and on the report of pain, associated with imaging examination, fracture of the body of the right maxilla and a complex fracture of the anterior arch of the C1 cervical vertebra were diagnosed.

**Interventions:**

In this case, we present a patient with trauma to the face and spine who was intubated through the nasopharyngeal route while awake and guided by video laryngoscopy and using a rigid cervical collar. The patient was operated on under total general anesthesia (propofol and remifentanil) and plates and screws were placed for maxillary osteosynthesis. The pain was alleviated with a peripheral block of the trigeminal nerve of the maxillary branch with 0.5% levobupivacaine.

**Outcomes:**

The patient woke up from surgery, was extubated uneventfully and without pain. Cervical spine injuries were followed up by the neurosurgery team for conservative treatment.

**Lessons:**

Patients with neck injury and facial trauma may need a definitive airway either for emergencies or for elective procedures. Intubating the awake patient may be an option when the anatomy of the cavity is unknown, and inducing the anesthetic act without this knowledge may be a inappropriate option, due to the risk of intubation/ventilation difficulties.

## 1. Introduction

Direct laryngoscopy can sometimes have limitations during the intubation procedure, especially in situations such as cervical immobility due to trauma. Reports show that 1/3 of all anesthetic deaths are due to failure to intubate and ventilate and that awake intubation may be an important alternative to be considered, as during this procedure, an open airway and spontaneous breathing are maintained until the point of protecting the airway, and the life-threatening scenario “cannot intubate, cannot ventilate” reduces the incidence.^[1,2]^

Furthermore, immobility of the cervical spine makes conditions worse for the procedure. Though several devices have been proven to be beneficial, little information is available on nasotracheal intubation in awake patients with cervical spine immobilization.^[3]^

## 2. Case presentation

A 41-year-old man (height: 171 cm; body weight: 68 kg), without previous comorbidities (American Society of Anesthesiologists I), was admitted to the emergency department of the reference hospital in traumatology brought by the mobile emergency service with a gunshot wound to the head and neck region. The patient was admitted and evaluated by the team using the advanced trauma life support protocol.

In the initial evaluation, the patient had patent airways, no signs of laryngeal stridor, was conscious and oriented, and with a rigid cervical collar. No changes in breathing (letter B in the assessment) or circulation (letter C in the assessment). On neurological examination, he was conscious and oriented in time and space, with isochoric and photoreactive pupils and without focal neurological deficits. He complained of pain in the face, mainly in the right maxilla and cervical region. Entry vital signs: blood pressure: 123/78 (93) mm Hg; pulse rate: 78; peripheral oxygen saturation: 97% on room air; respiration rate: 16; body temperature: 36.8ºC.

**Figure 1.**
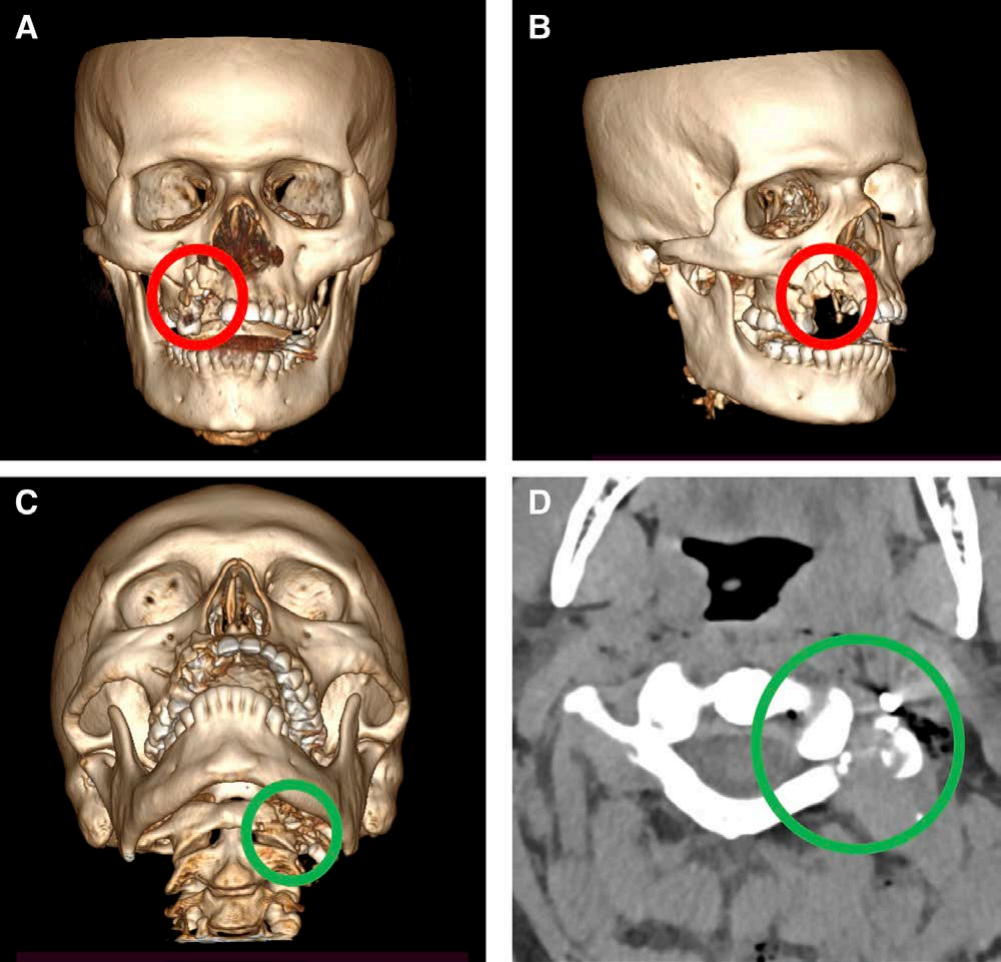
Preoperative multi-detector computed tomography. (A and B) Bone reconstruction in 3 dimensions showed a fracture of the body, lateral and inferior wall of the maxillary sinus on the right - red circle. (C) Bone reconstruction in 3 dimensions showed complex fracture was observed in the anterior arch, lateral mass, and in the left transverse process of the C1 cervical vertebra. (D) Coronal section of C1 cervical vertebra showing fracture with bone fragments and lodged bullet with light enhancement.

The patient underwent computed tomography without contrast of the skull and cervical spine 20 minutes after the initial evaluation. The examination showed a fracture of the body, lateral and inferior wall of the maxillary sinus on the right (Fig. 1A and B, red circle). In addition, a complex fracture was observed in the anterior arch, lateral mass, and in the left transverse process of the C1 cervical vertebra (Fig. 1C and D, green circle).

After evaluation by the maxillofacial surgery team, they opted to approach the patient in a surgical center and stabilize the maxillary fracture. The patient was taken to the operating room and due to limited head extension (using a rigid cervical collar), in addition to other predictors of difficult airway or ventilation difficulties, it was decided by awake nasotracheal intubation. He was pre-oxygenated with 100% O_2_, prophylaxis with Omeprazole 40 mg and Metoclopramide 10 mg, both intravenous. The nasotracheal intubation procedure was performed through the left nostril using lidocaine gel (20 mg/g) to pass the tube through the nostrils and 10% lidocaine spray with 6 puffs performed in the oropharynx and epiglottis region using the atomizer. A tied 7.0 endotracheal tube was used, guided by C-MAC D-Blade video laryngoscope and the aid of a Magill forceps, confirmed by capnography. After passing the tube, the patient remained stable and pain-free. He was induced with midazolam 3 mg; fentanyl 5 mcg/kg, propofol 2 mg/kg; rocuronium: 0.6 mg/kg). He remained under total intravenous anesthesia with propofol (maximum dose: 100 mcg/kg/minute) and remifentanil (maximum dose: 0.3 mcg/kg/minute), both in the continuous infusion pump. The pain was alleviated with a peripheral block of the trigeminal nerve of the maxillary branch with 0.5% levobupivacaine after surgical.

After the procedure for fixing the fractures with total duration of the 3 hours 54 minutes, airway aspiration was performed and extubation was performed uneventfully. He presented an Aldrete scale = 9 and was referred to the postanesthesia recovery room.

## 3. Discussion

One of the challenges of anesthesiologists is the management of patients with difficult airways. Today, one of the safest options is intubation with the patient awake using a device with video, either videolaryngoscopy or nasofibroscope. However, the use of a cervical collar in patients with spinal injuries makes the environment for intubation a real challenge, as it reduces the mouth opening and impairs the alignment of the acoustic meatus with the sternum.^[1,3]^

In this context, to avoid problems during the procedure and in case of failure of not being able to intubate or worse, not being able to ventilate, awake intubation helps to avoid situations that can cause sequelae if the patient becomes hypoxic.^[1]^

In the studies carried out by Seo et al,^[3]^ it was demonstrated that the use of a video laryngoscope was associated with an extended visualization of the vertical plane of the glottic areas in patients with the use of a cervical collar for oropharyngeal intubation, without other associated facial injuries. It is interesting to note that nasotracheal intubation by video laryngoscopy and using a cervical collar does not require alignment of the oral, pharyngeal and tracheal axes, in addition to reducing intubation time and reducing the need for additional maneuvers, such as tube rotation and backward, upward, and rightward pressure maneuver.^[3,4]^

Associated with this, patients with facial trauma, mainly of the maxilla or manila, also have limitations, as the force used to obtain a good glottal view in direct laryngoscopy can worsen the fracture, when compared to video laryngoscopy.^[3]^ In addition, by exerting less force, video laryngoscopy results in less injury to the oropharyngeal tissue and it is believed that this may reduce the hemodynamic response during the procedure. This makes awake nasopharyngeal intubation guided by video laryngoscopy in patients with facial and cervical spine fractures an excellent method to ensure airway patency in extensive surgical procedures.^[3,4]^

In addition, topical anesthesia is essential for successful nasotracheal intubation, thus reducing patient discomfort without compromising the airways. Kumar et al^[1]^ published interesting data that topical anesthesia with 4% lidocaine facilitated nasotracheal intubation in patients with maxillary fractures, in addition to reducing laryngeal trauma and the time required for the procedure. No different, we obtained results similar to the case report published by Saini et al,^[5]^ in which topical anesthesia with 10% lidocaine spray was used and intubation was successful.

Thus, the present report may help in decision-making in patients with associated fractures of the face and cervical spine and with predictors of difficult airway.

## Author contributions

Conceptualization: Luan Oliveira Ferreira, Victoria Winkler Vasconcelos.

Data curation: Imaikon Gomes de Lima, Juliana Barbosa de Souza.

Investigation: Victoria Winkler Vasconcelos.

Writing – original draft: Luan Oliveira Ferreira.

Writing – review & editing: Imaikon Gomes de Lima, Juliana Barbosa de Souza, Karina Dias Resende.
